# Optimization of the photoelectrocatalytic oxidation of landfill leachate using copper and nitrate co-doped TiO_2_ (Ti) by response surface methodology

**DOI:** 10.1371/journal.pone.0171234

**Published:** 2017-07-03

**Authors:** Xiao Zhou, Shaoqi Zhou, Xinbin Feng

**Affiliations:** 1 College of environmental science and engineering, Guangdong University of Technology, Guangzhou Higher Education Mega Center, PR China; 2 Institute of biology, Guizhou Academy of Sciences, Guiyang, PR China; 3 State Key Laboratory of Subtropical Building Sciences, South China University of Technology, Guangzhou, PR China; 4 Institute of geochemistry, China academy of sciences, Guiyang, PR China; 5 College of Environment and Energy, South China University of Technology, Guangzhou Higher Education Mega Center, PR China; 6 Key Laboratory of Environmental Protection and Eco-remediation of Guangdong Regular Higher Education Institutions, South China University of Technology, Guangzhou Higher Education Mega Center, PR China; Argonne National Laboratory, UNITED STATES

## Abstract

In this paper, a statistically-based experimental design with response surface methodology (RSM) was employed to examine the effects of functional conditions on the photoelectrocatalytic oxidation of landfill leachate using a Cu/N co-doped TiO_2_ (Ti) electrode. The experimental design method was applied to response surface modeling and the optimization of the operational parameters of the photoelectro-catalytic degradation of landfill leachate using TiO_2_ as a photo-anode. The variables considered were the initial chemical oxygen demand (COD) concentration, pH and the potential bias. Two dependent parameters were either directly measured or calculated as responses: chemical oxygen demand (COD) removal and total organic carbon (TOC) removal. The results of this investigation reveal that the optimum conditions are an initial pH of 10.0, 4377.98mgL^-1^ initial COD concentration and 25.0 V of potential bias. The model predictions and the test data were in satisfactory agreement. COD and TOC removals of 67% and 82.5%, respectively, were demonstrated.

Under the optimal conditions, GC/MS showed 73 organic micro-pollutants in the raw landfill leachate which included hydrocarbons, aromatic compounds and esters. After the landfill leachate treatment processes, 38 organic micro-pollutants disappeared completely in the photoelectrocatalytic process.

## Introduction

Leachate produced from landfills is highly polluted wastewater presenting intense and persistent toxicity, which has been a cause for great concern because dumping is the most routine method in solid waste disposal. The composition and concentration of pollutants are persuaded by the sort of waste and the age of the disposal area[1]. Leachates may contain sizeable amounts of both biodegradable and resistant organic matter, with a predominance of humic substances[2], heavy metals, chlorinated organic compounds and inorganic salts[3]. If poorly collected and treated, landfill leachate may become a source of pollution due to the infiltration of the leachate into ground and surface waters[4]. In the past, the most popular remedies of landfill leachate were organic treatments[5]. However, organic treatments are not entirely efficient in degrading the refractory organic pollutants and in decoloring the leachate. Furthermore, degradation efficiency depends on changeable organic loads and various flow rates. Hence, the advanced oxidation process (AOP) of landfill leachate either as a post or pre-treatment to improve its biodegradability and treatability has attracted a great deal of interest[6]. As one of the progressive oxidation method technologies for leachate treatment, photoelectrocatalysis (PEC) has received increasing attention in the area of environmental treatment due to its ability to destroy refractory organic compounds and to remove traces of organic species[7]. In 1982, Ward et al. combined TiO_2_ film with a positive cathode; with this development based on photocatalytic technology[8], PEC has been used in environmental preservation studies. The photoelectrocatalytic technique combines electrolytic and photocatalytic processes. The PEC process involves applying an electrical bias to a photocatalytic process to stop the reunion of electron-hole pairs ($e_{CB}^{-}/h_{VB}^{+}$) with the possibility of increasing their lifetime. Generally, under the strength of the electrical bias, the $e_{CB}^{-}$ voluntarily moves to the outside circuit and concentrates in the cathode. The lack of electrons causes the assembly of holes in the anode, which equally leads to the sought after reduction of the recombination rate. The apertures on the exterior of the anode could either immediately oxidize some organic genus or react with the adsorbed water to create hydroxyl radicals, which can thoroughly break down the refractory organics. The electrons assembled in the cathode could also react with the dissolved oxygen to form superoxide radicals[9].

Until now, statistical planning tools have not been used to standardize and improve PEC for various original pollutant concentrations (organic pollution loads) or for setting numerous targets to reach treatment capabilities at various levels, depending on legislative or other obligations. Response surface methodology (RSM) is an assortment of statistical and mathematical methodologies that can include the effects of unique factors, as well as their interactive effects. RSM is employed to solve multivariable equations and to simultaneously evaluate the comparative importance of several affecting factors, even in complicated systems, by multiple regression analysis using quantitative data collected from appropriately designed tests[10]. Compared to classical experimental optimization methods, which are characterized by a “single process variable at a time” technique, using the RSM design can decrease the number of tests the time required. In addition, the ultimate goal of RSM is to explore the area of the response surface near the ideal or to ascertain the ideal operating environment for the system, thus determining the best conditions to obtain the desirable responses[11]. Analysis of variance (ANOVA) supplies the statistical results and diagnostic tests which allow researchers to evaluate the competence of the models[12]. For the past few years, many studies have proved that RSM could serve as a potent statistical tool for optimization of method parameters[13]. RSM use with innovative oxidation processes has been published by many research organizations [14–16]. The RSM approach has also been used to optimize and evaluate the interactive effects of independent factors in photocatalytic degradation studies for several types of pollutants[17–23] such as azo dyes (e.g., Reactive Red 120 and Reactive Red 239)[24], phenol aqueous, chloramphenicol aqueous [25] and natural organic matter(NOM)[26]. However, the effect of the interaction of different operating conditions on the organics elimination efficiency using RSM methodology during the photoelectrocatalytic oxidation process has not been reported.

Based on the preferred features of orthogonality and ability to rotate, central composite design (CCD) and box-benkhen design (BBD) are generally used for response optimization [11]. CCD is the most often used five-level fractional factorial design for the creation of second-order response surface models. Considering that the photoelectrocatalytic oxidation process is an energy-intensive process, the photoelectrocatalytic oxidation of the landfill leachate from reverse osmosis was investigated and optimized via CCD.

To our knowledge, the optimization of the operation parameters of photo- electrocatalytic oxidation landfill leachate using copper and nitrate co-doped TiO_2_ (Ti) as the photo-anode via CCD has not been reported. For that purpose, a range of photoelectrocatalytic oxidation trials were first run to identify the test design range in the current study. Using a CCD model of RSM, the photoelectrocatalytic treatment of landfill leachate rejected by reverse osmosis (RO) performance was first appraised in terms of COD and TOC elimination efficiency for the optimization of the key factors, such as electrical bias, pH, and COD abundance. The effects of the initial pH, electrical bias and the COD concentration among these parameters on the oxidation achievement to remove organics were studied in this evaluation. Quadratic models were employed to adjust the studied experimental results, since only the variables that presented significant effects. Two interrelated factors, such as COD removal and TOC removal were appraised as responses. Moreover, a continuous response surface of the main parameters was created to contribute an ideal area to satisfy the operating requirements. This study develops a standard model for the landfill leachate deterioration rate engaging three separate factors, optimizes the degradation process of leachate under the related constraint conditions and provides a new method to address landfill leachate pollution. The micro-organic components were also studied by gas chromatography-mass spectrometry (GC/MS).

## Materials and methods

According to PLoS ONE submissions requirements for field studies. For this location of landfill site for which specific permission was not required, we declare that no specific permissions were required for these locations or activities, and we did our study at our lab which in College of Environment & Energy, south china university of technology, we did all the work in accordance with the regulations. Therefore, the specific permissions are not necessity. Also, we confirm that the field studies did not involve endangered or protected species.

### Materials

Concentrated leachate returned from RO was gathered from the Guangzhou Domestic landfill site (600 m^3^/d), South China, where the merged processes of up-flow anaerobic sludge blanker (UASB), sequencing batch reactor (SBR), continuous micro-filtration (CMF), and reverse osmosis(RO) were applied successively. The samples were stored in the dark, and at 4°C and in the dark. The average configuration of the tested undiluted leachate effluent returned from RO was as follows: pH 7.8, COD (4378 mg/L), DOC (2583 mg/L), BOD_5_ (29.1 mg/L), BOD_5_/COD 0.01, HS (1337.4 mg/L), electrical conductivity (39.2 ms/cm). The correlation of BOD_5_ to COD was approximately 0.01, which suggested that the concentrated leachate is difficult to correct biologically. The raw undiffused landfill leachate was screened through a 0.45μm glass-fiber filter to expel large fragments and debris and to maintain the regularity of tested samples.

A graphite electrode was purchased from Guangzhou Jinlong Technology Co., Ltd. The photoelectrocatalytic oxidation was performed at a constant current using a digital DC power supply. Titanium sheets (0.2 mm×50 mm×50 mm, 99.6% purity) were polished using grinding paper and then degreased using ultrasonication in acetone, isopropanol and methanol. After rinsing with water, the materials were air-dried. Acetone, isopropanol, methanol, HF, HNO_3_, Cu(NO_3_)_2_, NH_4_Cl, and the above-mentioned chemicals and solvents were of technical grade and were not purified prior to use.

### Instruments

The COD and BOD_5_ measurements were taken according to standard methods (APHA, 2005); the DOC of the reagent was measured with a liquid TOC analyzer (Germany); the sample solution pH was measured using a pH meter (pHS-25C, Jingke Co.LTD, Shanghai); and the electrical conductivity was analyzed using a conductivity meter (DDS-11A, Leici Co. LTD, Shanghai). A 50 W tungsten halogen lamp (EXZ MR16 SP, GE, USA) was used as a visible light source. Constant temperature water bath equipment (HH-501, Jingfeng Co, LtD, Shanghai) was used to control the required temperature during the reaction.

### Preparation of photo-anode

A sheet of commercial titanium was doped with copper and N elements in 2:3 molar ratios of Cu^2+^/NH_4_^+^ using anodic oxidation technology to prepare Cu_2_N_3_ co-doped TiO_2_/Ti. First, the titanium sheet was placed in the electrolyte containing 0.01 M HF and 0.1 M HNO_3_ under a certain voltage for a certain time. Then, the as-prepared TiO_2_ electrode (on the Ti sheet) was placed in an electrolyte solution of 0.4 M Cu(NO_3_)_2_ and 0.6 M NH_4_Cl under a certain voltage for a certain time. The titanium sheet with a Cu_2_N_3_ co-doped TiO_2_ surface was rinsed with water and air-dried. Then, the Cu/N-co doped TiO_2_ electrodes were calcined at 500°C under air for 2 h in a muffle furnace.

### Degradation experiments

The testing was conducted in a batch reactor made up of a 1000 mL borosil beaker above a magnetic stirrer. The magnetic maintained at a minimal speed during the entire process.

A 50 W tungsten halogen lamp (EXZ MR16 SP, GE, USA) emitting a wavelength range from 380 nm to 780 nm served as the visible light source. The light intensity was 80.1mWcm^-2^(380nm~780nm) measured by spectrascan spectroradiometers (PR-705, Photo Research, USA). The degradation was tested under pH values of 2, 4, 6, 8 and 10, an electrical bias of 5 V, 10 V, 15 V, 20 V and 25V was used. An initial COD concentration ranging from 876 mgL^-1^ to 4378 mgL^-1^ was employed. Constant-temperature water-bath equipment (HH-501, Jingfeng Co, LTD, Shanghai) was used during the reaction to maintain the desired temperature. The initial leachate pH was corrected to the desired value with concentrated sulfuric acid and sodium hydroxide.

### Analysis

The degree of mineralization of the leachate was determined by measuring the COD disposition at different time intervals employing a standard method with potassium dichromate. The COD disposition was resolved using Eq (1):

$$COD\;removal\;(\%)\;=\left(\frac{COD_{0}-COD_{t}}{COD_{0}}\right)\times 100$$ (1)

Where *COD*_*0*_ is the initial COD in mg/L, and *COD*_*t*_ is the COD in mg/L at any time *t*.

$$TOC\;removal\;(\%)\;=\left(\frac{TOC_{0}-TOC_{t}}{TOC_{0}}\right)\times 100$$ (2)

The TOC sample values were measured with a TOC analyzer (Shimadzu, Germ any). The identification and approximate concentrations of organic micro-pollutants were detected using GC-MS analysis (Agilent 7890A-5975C, USA). The temperature ramp for the GC/MS was as follows: 60°C for 10min, 50–220°C at 5.0°C min^-1^ and 220°C held for 10min and 220~290°C at 5.0°C min^−1^ and 290°C held for 5min. The extracts were prepared according to EPA test method 625 based on liquid–liquid extraction with methylene chloride. The extract was dried by filtering it through a column of sodium sulphate and concentrated with a rotary evaporator (RE-52A, China).

### Experimental design and statistical analysis

To identify the optimum conditions for deterioration of the leachate and to reach sufficient and reliable measurements of the reactions of interest, the experimental strategy was determined. Design Expert Software (version 8.0) was used for the statistical strategy of the testing and data analysis. In this study, we adopted a five-level full-factorial CCD, which is an effective design instrument for fitting second-order models to optimize the photoelectrocatalytic reaction parameters. The three selected experimental parameters chosen in this study are pH, electrical bias and initial COD concentration, which was improved using RSM considering them as separate variables and considering TOC and COD elimination as the response variables. As demonstrated in Table 1, this rotatable experimental plan was performed as a CCD consisting of 20 experiments. All of the variables were captured at a central coded value of zero. Each parameter in the design was studied at five different levels (−2, −1, 0, 1, 2).

**Table 1:** The observed and predicted COD and TOC elimination efficiencies using the CCD model.

Run							Observed		Predicted	
	*x*_1_	*x*_2_	*x*_3_	*X*_1_ pH	*X*_2_ COD concentration	*X*_3_ electrical bias	COD removal	TOC removal	COD removal	TOC removal
1	-1	-1	-1	4	1752	10	60.235	46.67	60.32684	47.14
2	1	-1	-1	8	1752	10	50.341	34.8	49.99832	33.88
3	-1	1	-1	4	3503	10	40.121	16.3	43.50989	17.76
4	1	1	-1	8	3503	10	39.204	17.7	34.4087	10.87
5	-1	-1	-1	4	1752	10	66.729	48.2	73.22858	56.87
6	1	-1	-1	8	1752	10	59.711	37.26	62.78148	46.35
7	-1	1	-1	4	3503	10	58.681	38.9	60.96777	44.91
8	1	1	-1	8	3503	10	51.865	35.46	51.74801	40.74
9	-1	-1	1	4	1752	20	74.333	59.92	73.22858	56.87
10	1	-1	1	8	1752	20	68.641	58.1	62.78148	46.35
11	-1	1	1	4	3503	20	62.312	51.2	60.96777	44.91
12	1	1	1	8	3503	20	51.123	47.7	51.74801	40.74
13	-2	0	0	2	2627	15	78.859	60.49	74.24972	56.26
14	1	-1	1	8	1752	20	50.692	33.4	54.70143	38.84
15	-1	1	1	4	3503	20	70.466	55.2	69.58829	53.34
16	1	1	1	8	3503	20	41.46	15.3	41.73786	18.36
17	-2	0	0	2	2627	15	48.235	23.12	49.54107	28.96
18	2	0	0	10	2627	15	32.556	14.78	33.68455	17.09
19	0	-2	0	6	876	15	65.654	57.8	63.92559	56.7
20	0	2	0	6	4378	15	51.447	33.6	49.54107	28.96

The test data was analyzed using the RSM method of the statistical analysis system and equipped with a second-order polynomial equation using a multiple regression technique as follows:

$$Y=\beta_{0}+\sum \beta_{i}X_{i}\text{+}\sum \beta_{ii}X_{i}^{2}\text{+}\sum_{i}\sum_{j}\beta_{ij}X_{i}X_{j}\text{+}\cdots +\;e$$ (3)

where *Y* is a response variable of decolorization efficiency, *β*_0_ is a constant coefficient; *β*_*i*_ is the regression coefficients for linear effects; *β*_*ii*_ is the regression coefficients for quadratic effects for the factors, *i* and *β*_*ij*_ the linear model coefficient for the interaction between factors *i* and *j*. *X*_*i*_ is the coded experimental level of the primary parameters. In the present study, ANOVA and response surface plots were performed using Design Expert Software (version 8.0, Stat-Ease, Inc., Minneapolis, USA), which was used to estimate the coefficient parameters of the second-order models by a multiple linear regression analysis.

ANOVA for the model was performed for graphical analyses to determine the statistical importance and dependability of the data. The quality of the fit of the polynomial sample was expressed by the coefficient of determination *R*^*2*^ and Adj-*R*^*2*^. The *R*^2^ values quantify the amount of difference in the observed response values that can be clarified by the experimental factors and their reactions. The *R*^2^-value is always between 0 and 1. The closer the *R*^2^-value is to 1, the better the model predicts the response[27]. The significance was analyzed with the Fisher variation ratio (F-value) in the polynomial equation. The model terms were decided upon based on the *P* value (probability) with a 95% assurance level.

Three-dimensional plots and their respective contour plots were acquired based on the results of the three factors at five levels. In addition, the perturbation plot was adopted to correlate the effect of all of the components at a particular point in the design area. Additionally, a comparison of the experimental data with predicted values obtained from the equations could verify the adequacy of the regression formula. A detailed investigation of the model is presented in this paper.

## Results and discussion

### Establishment of the experimental design matrix using factorial design

To study the effect of independent process variables on the responses over the investigated range and for the response surface modeling and optimization of the PEC, all 20 test runs of the CCD were completed in arbitrary order, which included 16 factorial points, 2 center and 8 axial points, and for each test run, the percent COD and TOC elimination efficiencies were determined (Table 1). As shown in Table 1, the CCD consists of three independent variables-*X* ((*X*_*1*_(pH); *X*_*2*_ (COD concentration) and *X*_*3*_ (electrical bias))—at five levels: -2 (minimum), -1, 0, 1, +2 (maximum), and the response *Y* (% of COD elimination (*Y*_*1*_) and % of TOC elimination (*Y*_*2*_)). The separate factors and their ranges were selected based on the preliminary test results. Table 1 shows the coded and actual values of the critical parameters used in the tests to calculate the response variables of COD removal (% *Y*_*1*_) and TOC removal (% *Y*_*2*_).

The witnessed COD elimination efficiencies changed between 32.56% and 78.86%; the TOC removal efficiencies ranged from 14.78% to 60.49% after 180 min oxidation. The maximum COD and TOC removal effectiveness was found to be 89.3% and 60.49%, respectively. Most often the observed and predicted results were in good agreement. These results were accomplished under the test conditions of *X*_*1*_ (pH 2), *X*_*2*_ (2627 mg/L COD concentration) and *X*_*3*_ (15 V electrical bias).

Table 2 presents the regression equations of the fitted models for PEC which were obtained from the analysis of variances. The quadratic equation determined for the COD and TOC elimination efficiency indicates the intensity and direction of the influence of the independent variable. The product of the independent variable can be directly assigned to the value of its coefficient [11]. Additionally, factors that exert a more pronounced effect have higher absolute values. The coefficients of the independent variable and its algebraic sign appear to evaluate the relative effect of each variable on the COD and TOC elimination efficiencies.

**Table 2:** Regression equations obtained for COD removal (*Y*_*1*_) and TOC (*Y*_*2*_) removal (%) of landfill leachate.

Analysis	Regression equations	
Analysis in coded factor (*x*_*1*_, *x*_*2*_, *x*_*3*_)	$\begin{array}{l} COD(Y_{1})=49.54-4.89x_{1}-6.96x_{2}+7.56x_{3}+0.31x_{1}x_{2}\\ -0.030x_{1}x_{3}+1.14x_{2}x_{3}+\;3.73x_{1}^{2}+1.53x_{2}^{2}-0.18x_{3}^{2} \end{array}$	(4)
	$\begin{array}{l} TOC(Y_{2})=28.96-4.35x_{1}-8.75x_{2}+9.90x_{3}+1.59x_{1}x_{2}+0.68x_{1}x_{3}\\ +4.35x_{2}x_{3}+\;4.65x_{1}^{2}+1.72x_{2}^{2}+1.98x_{3}^{2} \end{array}$	(5)
Analysis in uncoded factor (*X*_*1*_, *X*_*2*_, *X*_*3*_)	$\begin{array}{l} COD(Y_{1})=120.89-14.06x_{1}-0.023x_{2}+1.07x_{3}+1.75\text{E}-4x_{1}x_{2}-2.96E\\ -3x_{1}x_{3}+2.60E-4x_{2}x_{3}+\;0.933x_{1}^{2}+2.0E-6x_{2}^{2}-7.36E-3x_{3}^{2} \end{array}$	(6)
	$\begin{array}{l} TOC(Y_{2})=28.96-4.35x_{1}-8.75x_{2}+9.90x_{3}+1.59x_{1}x_{2}+0.68x_{1}x_{3}\\ +4.35x_{2}x_{3}+\;4\text{.65}x_{1}^{2}+1.72x_{2}^{2}+1.98x_{3}^{2} \end{array}$	(7)
	For −2 ≤ *X*_*i*_ ≤ 2	

Based on the coefficients given in Eqs. (4–7), the variable electrical bias (*x*_*3*_,*X*_*3*_*)* showed the highest positive influence on the COD and TOC elimination efficiencies. The COD and TOC elimination efficiencies increase in relation to the electrical bias (*x*_*3*_, *X*_*3*_), i.e., increasing the electrical bias increases the photoelectrocatalytic oxidation efficiency. Upon further examination of the polynomial regression model acquired for the COD and TOC removal, it is evident that the initial COD concentration possessed the largest effect on the COD and TOC elimination efficiency, and the “-” sign suggests that this effect is negative; thus, increasing the initial COD concentration results in reduced mineralization rates. The negative effect of the pH and COD concentration was also found to be significant. The COD removal efficiency decreases with pH and with a more profound effect of the COD concentration, whereas the pH had the smallest effect among the entire studied independent process variables. Additionally, the TOC removal efficiency decreases with the pH while decreasing with COD concentration. Generally, the observation that the coefficients for the process dependent variable TOC are higher than for the variable COD can be assigned to the fact that TOC represents the utmost oxidation, which is more challenging to achieve than that of the origin compound or COD reductions [16]. A positive factor effect is an improved response when the factor level increases, and a negative factor effect is an inhibited response when the factor level increases.

Table 3 contains the results of the quadratic response surface model fitting in the form of ANOVA, which is employed to check the importance and sufficiency of the model. The F-value (Fisher variation ratio), probability of error (Prob>F) and adequate precision (AP) are the main indicators of the importance and sufficiency of the employed model.

**Table 3:** ANOVA results for the response surface quadratic model for a 180-min PEC process.

Response	*F*	*P*	*LOF*	*PLOF*	*R*^*2*^	*Adj- R*^*2*^	*AP*	*SD*	*CV*	*PRESS*
*Y*_*1*_	17.67	<0.0001	1.19	0.4263	0.9408	0.8876	13.634	4.21	7.50	773.65
*Y*_*2*_	7.2	0.0024	0.28	0.9048	0.8663	0.7460	8.199	7.93	20.19	2154.29

P: probability of error, LOF: lack of fit F-value, PLOF: probability of lack of fit, R^2^: determination coefficient, Adj. R^2^: adjusted R^2^, AP: adequate precision, SD: standard deviation, CV: coefficient of variation, PRESS: predicted residual error sum of squares

In Table 3, the F-value was calculated by dividing the model mean square by the residual mean square. The models (*Y*_*1*_,*Y*_*2*_) F-values of 17.67 and 7.2 with a very small probability value (P < 0.0001 and 0.0024, respectively) revealed that the terms were significant in the model. There was only a 0.01% and 0.24% probability that the model’s F-value was attributed to noise. Thus, all of the factors, with respect to the main interactions and quadratic terms are meaningful. Because values of P< 0.0500 indicated that the model is important, the values greater than 0.1000 are usually considered unimportant [28].

The lack of fit (LOF) F-values describing the diversity of the data around the fitted model were insignificant in relation to the pure error. The model’s (*Y*_*1*_,*Y*_*2*_) LOF F-values of 1.19 and 0.28 indicated that the lack of fit is insignificant in relation to the pure error. There was 42.63% and 90.8% probability for *Y*_*1*_ and *Y*_*2*_, respectively, that the LOF F-value was attributed to noise. The value of the likelihood of lack of fit (PLOF) >0.05 indicates that the F-value was insignificant, indicating a significant model relationship between the variable and method response. If the model does not fit the data well, then this will be important.

Table 3 also shows the coefficient of determination (*R*^*2*^) that reveals whether the data were fitted well by the polynomial regression models. The *R*^*2*^*-*values give the percent variability in the response demonstrated by the statistical model. In this study, high *R*^*2*^ values ranging from 0.9408 to 0.8663 were obtained for *Y*_*1*_ and *Y*_*2*_. The *R*^*2*^*-*value of the response variables conformed to the order of *R*^*2*^ (COD) > *R*^*2*^ (TOC). The highest *R*^*2*^-value obtained for the COD elimination efficiency suggests that 94.08% of the total variation could ensure an acceptable adjustment of the quadratic models to the test data. The smaller the *R*^2^-value, the less relevant the model fits the actual data. Research [29] showed that the *R*^2^ -value should be at least 0.80 for a model to have a good fit. The edited *R*^2^ (Adj *-R*^2^) corrects the *R*^2^-value for the sample size and the number of terms in the model. The Adj-*R*^*2*^ values were 0.8876 and 0.7460 for *Y*_*1*_ and *Y*_*2*_ of the models, respectively. If there are a large number of terms in the model and the sample size is small, then the Adj-*R*^*2*^ may be conspicuously smaller than *R*^2^. However, when the *R*^*2*^-value is close to 1, it is in sound agreement with Adj-*R*^*2*^.

The AP parameter equates the range of the predicted values at the design points to the average prediction error. AP values >4 indicate sufficient model distinction and that the two predicted models can be used to follow the design space defined by CCD[30]. Additionally, the coefficient of variance (CV), which is usually the ratio of the standard error of the estimate to the mean value of the observed response, defines the ability to reproduce the model. Typically, if its CV is not greater than 10%, a model can be considered reproducible [31]. The CV-value (7.50%) for the *Y*_*1*_ model displayed acceptable precision and reliability in the experiments, and the *Y*_*2*_ model (CV = 20.19) which fails in terms of reproducibility, is the model for TOC removal.

Considering the ANOVA test results as explained above, the model application illustrated the reaction well and can be used to follow the design space in terms of COD and TOC elimination efficiencies.

As shown in Table 4, the mean squares were acquired by dividing the sum of the squares of both sources of variation, the model and the error (residual) variance. The response surface plots of the model-predicted responses keeping one variable constant and changing the others within the experimental ranges were proven. The *P*-values were used as a tool to determine the importance of each of the coefficients, which indicated the pattern of the interfacing between the test variables. The parameter valuation and the corresponding *P-*values indicate that, among the test variables, the synergistic effect of the linear term of the COD concentration (*X*_*2*_) and potential bias (*X*_*3*_) was highly significant for the responses of *Y*_*1*_ (*p*-value< 0.0001). The responses of *Y*_*1*_ were not very significantly affected by the reciprocal effect of the linear term of pH (*p*-value = 0.0005) and the contrary effect of the quadratic term of the pH (*X*_*1*_^*2*^) (p-value of 0.053). Although the effect of the initial COD concentration (*X*_*2*_) and the initial potential bias (*X*_*3*_) was more pronounced than the initial pH(*X*_*1*_), the favorable quadratic effect (*X*_*1*_^*2*^) indicates that the removal efficiency is enhanced at very low values. The most substantial effect of the linear term is still derived from the first COD concentration (*X*_*2*_) and the potential bias(*X*_*3*_).

**Table 4:** Results for the reduced cubic model of the variable effects on the response.

Source	Sums of squares		Mean squares		*F*		*P*	
	*Y*_*1*_	*Y*_*2*_	*Y*_*1*_	*Y*_*2*_	*Y*_*1*_	*Y*_*2*_	*Y*_*1*_	*Y*_*2*_
*X*_*1*_	445.82	353.86	445.82	353.86	25.18	5.62	0.0005	0.039
*X*_*2*_	904.92	1427.53	904.92	1427.53	51.11	22.67	<0.0001	0.0008
*X*_*3*_	1097.42	1882.78	1097.42	1882.78	61.99	29.91	<0.0001	0.0003
*X*_*1*_*X*_*2*_	1.13	30.37	1.13	30.37	0.06	0.48	0.8057	0.503
*X*_*1*_*X*_*3*_	*0*.*0098*	5.21	*0*.*00984*	5.21	0.0006	0.08	0.9817	0.779
*X*_*2*_*X*_*3*_	14.53	212.18	14.53	212.18	0.82	3.37	0.3863	0.096
*X*_*1*_^*2*^	223.04	345.4	223.04	345.4	12.6	5.49	0.0053	0.041
*X*_*2*_^*2*^	37.48	47.47	37.48	47.47	2.12	0.75	0.1763	0.406
*X*_*3*_^*2*^	0.54	62.88	0.54	62.88	0.03	0.99	0.8646	0.341
*Residual*	177.04	629.59	17.7	62.96				

*X*_*1*_, *X*_*2*_ and *X*_*3*_ represent the main effect of initial pH, COD concentration and potential bias, respectively. *Y*_*1*_: overall COD removal efficiency, *Y*_*2*_: TOC removal efficiency, *X*_*1*_*X*_*2*_, *X*_*1*_*X*_*3*_ and *X*_*2*_*X*_*3*_ represent the interaction between initial pH and COD concentration, interaction between initial pH and potential bias and interaction between COD concentration and potential bias, *X*_*1*_^*2*^, *X*_*2*_^*2*^ and *X*_*3*_^*2*^ represent the quadratic effect of initial pH, COD concentration and potential bias, respectively.

The linear relationships between the initial COD concentration and the TOC removal efficiency (*Y*_*2*_) and those between the potential bias and the TOC removal efficiency (*Y*_*2*_) were significant (*p*-values = 0.0008 and 0.0003, respectively), and the pH(*X*_*1*_) factor was not substantial, with high *P* values. The coefficients of the quadratic effects among the variables did not appear to be substantial in comparison to the linear effect for the TOC elimination efficiency. However, none of the variables were found to be significant in interaction effect, except between the initial COD concentration and the potential bias (*P* = 0.0963). Additionally, the quadratic terms of the pH (*X*_*1*_^*2*^) have a contrary effect on *Y*_*2*_ responses (*p*-values of 0.0412). These observations can be interpreted to be due to a corresponding relationship between the variables and the removal effectiveness. The conclusions suggest that the interactions of the other two parameters did not substantially enhance the removal efficiency.

### Response surface plots and optimization

**Fig 1:**
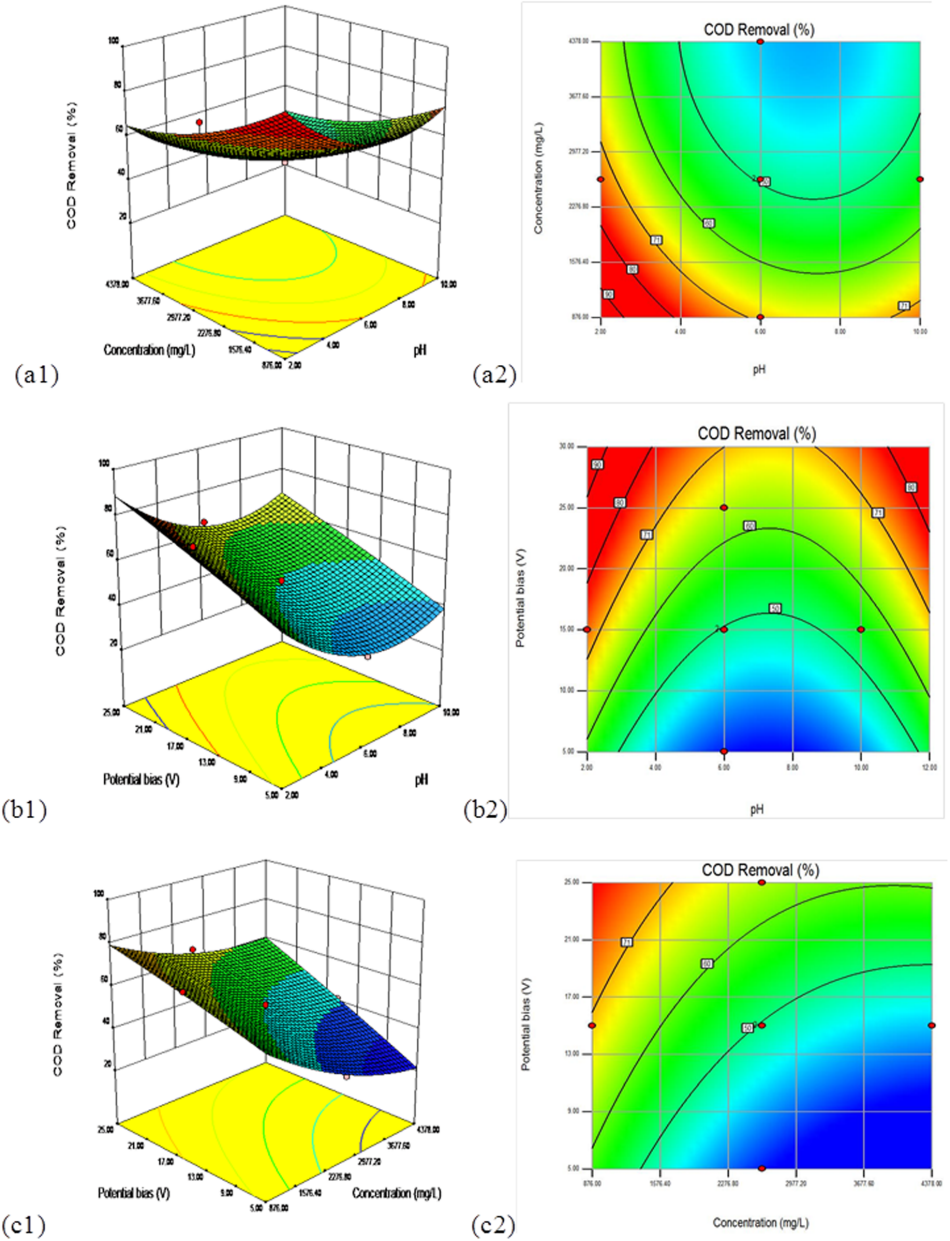
Surface and contour plots of COD removal efficiency in uncoded values for 180 min. (a) *X*_*1*_ (pH) and *X*_*2*_ (COD concentration) in fixed *X*_*3*_ (potential bias) at 15 V, (b) *X*_*1*_ (pH) and *X*_*3*_ (potential bias) in fixed *X*_*2*_ (COD concentration) at 2627 mg/L, (c) *X*_*2*_ (COD concentration) and *X*_*3*_ (potential bias) in fixed *X*_*1*_ (pH) at 6.

**Fig 2:**
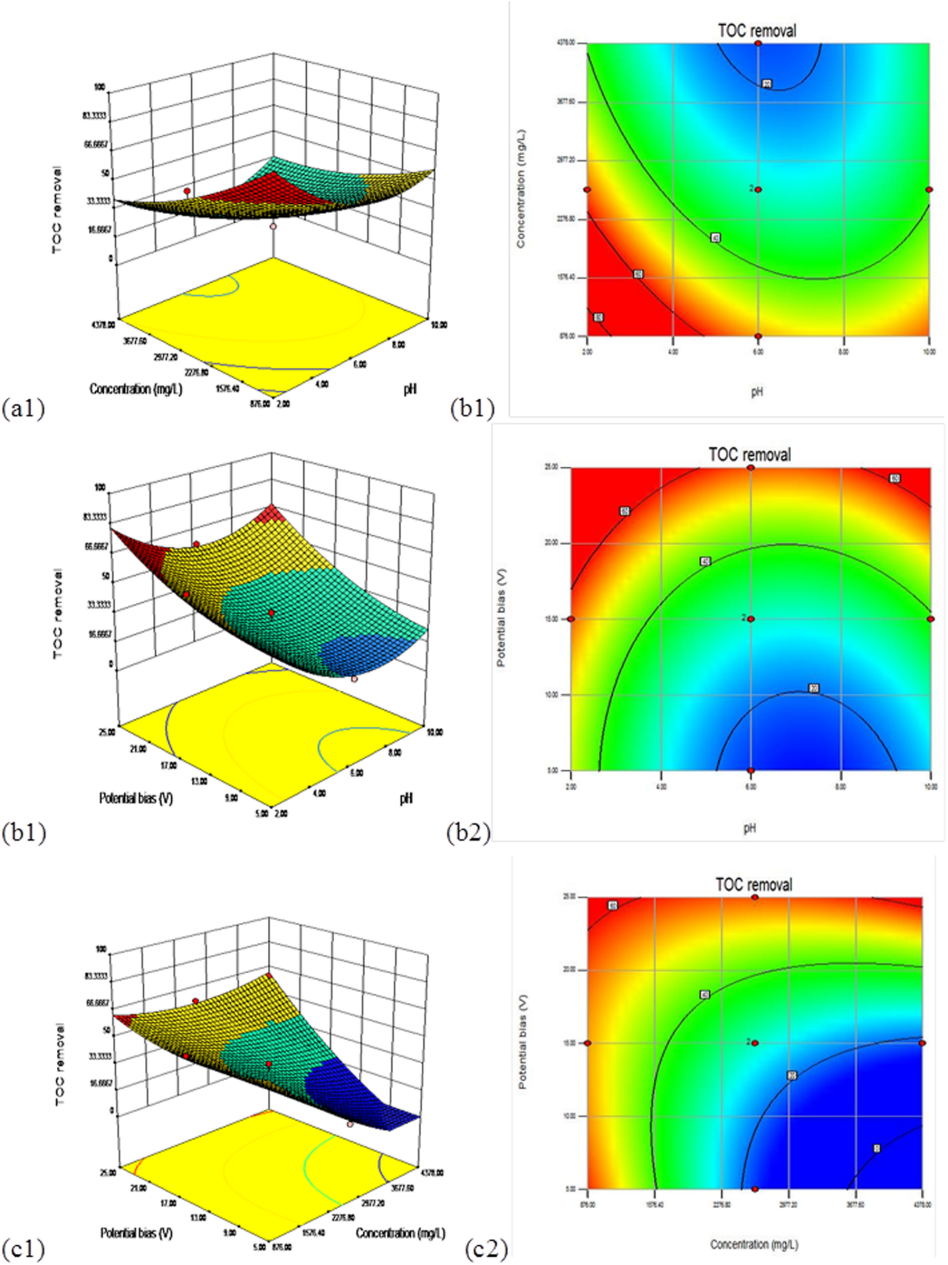
Surface and contour plots of TOC elimination efficiency in uncoded values for 180 min. (a) *X*_*1*_ (pH) and *X*_*2*_ (COD concentration) in fixed *X*_*3*_ (potential bias) at 15 V, (b) *X*_*1*_ (pH) and *X*_*3*_ (potential bias) in set *X*_*2*_ (COD concentration) at 2627 mg/L, (c) *X*_*2*_ (COD concentration) and *X*_*3*_ (potential bias) in set *X*_*1*_ (pH) at 6.

Employing RSM, three-dimensional (3D) and two-dimensional contour (2D) plots for the predicted responses (*Y*_*1*_,*Y*_*2*_) were also represented. To assist visualization and to help in determining the type of interactions between the test variables, the response surfaces for the COD elimination efficiency and mineralization are shown in Figs 1 and 2. The effects of the independent variables (pH, initial COD concentration, and potential bias) and their interaction for the COD removal and mineralization of the landfill leachate can also be further studied using these plots. A set of two response surface diagrams were created for each response(*Y*_*1*_,*Y*_*2*_) because the model has several factors, and one factor was used consistently in each diagram.

Figs 1 and 2 describe the minimum point for COD removal efficiency (*Y*_*1*_) response and TOC removal efficiency (*Y*_*2*_) and show graphical 3D and 2D illustrations of the polynomial acquired from the matrix.

Figs 1a and 2a depict the variation of the COD and the TOC removal efficiency, respectively, with pH under different COD concentration circumstances in the presence of a steady potential bias of 15V, respectively. From 1a, the effects of the pH and the initial COD concentration indicate that the increase of the COD and TOC elimination efficiency became relevant and gradual for [pH] < 6 and [initial COD concentration]< 2627 mg L^−1^. The greatest COD elimination efficiency was found at the concentration of 2627 mg L^−1^ and a pH of 2, as depicted in the contour plot. Fig 2a illustrated similar effects of the first landfill leachate concentration and initial pH on the mineralization rate as in the case of COD removal.

Figs 1b and 2b depict the effect of the pH and potential bias, while keeping the initial COD concentration at the middle level (2627 mg/L) for response surfaces related to COD and TOC removal efficiency, respectively. As seen from Fig 1b, the gain in the potential bias and the decline in pH increased the COD removal at pH 2 and potential bias ≥15 to attain a maximum value. This behavior is not surprising because at the optimal potential bias the electrons and holes are so well separated and accelerating the photocatalytic oxidation of organic pollutants. Fig 2b displayed comparable trends of the effects of the pH and potential bias on the decomposition and oxidation of landfill leachate as COD elimination, with the greatest region at pH 2~6 and potential bias 15~25 V.

The interaction results of the initial COD density and the initial potential bias with a constant pH of 6 on the COD removal efficiency are shown in Fig 1c, and the same for the mineralization of the landfill leachate are shown in Fig 2c. As seen in Fig 1c, the COD removal efficiency increased with the gain in potential bias, regardless of the initial COD density, with the maximum region in the potential bias range from 15 to 25 V. Similarly, the results showed a decline in the COD removal with the gain in the initial COD density. Fig 2c indicates that the TOC elimination efficiency grew with the gain in the potential bias setting along with the initial COD density of the landfill leachate, while it declined with the growth in the initial COD density with a peak concentration of 2627 mgL^-1^ and a potential bias of 25 V.

### Optimization of the independent variables

The main objective of the optimization is to ascertain the ideal values of the three independent variables for landfill leachate treatment regimen with the photoelectrocatalytic process from the two response models acquired using test data. Any response system is enhanced to determine an ideal operating environment that will elicit the optimal result using different design method and analytical methods. However, a good optimization procedure must consider the effects of components such as economy, ecological balance, and the likelihood of later treatment, in addition to magnifying the result. The ideal conditions for the greatest possible decomposition and oxidation of landfill leachate under the described restrictions were discovered to be an initial pH of 10.0 for the reaction mixture, 4377.98mgL^-1^ of the initial COD concentration and 25.0 V of potential bias. In this environment, the model anticipated that the COD removal and TOC removal of the landfill leachate as be 67.0% and 82.5%, respectively.

### Verification of the results

**Fig 3:**
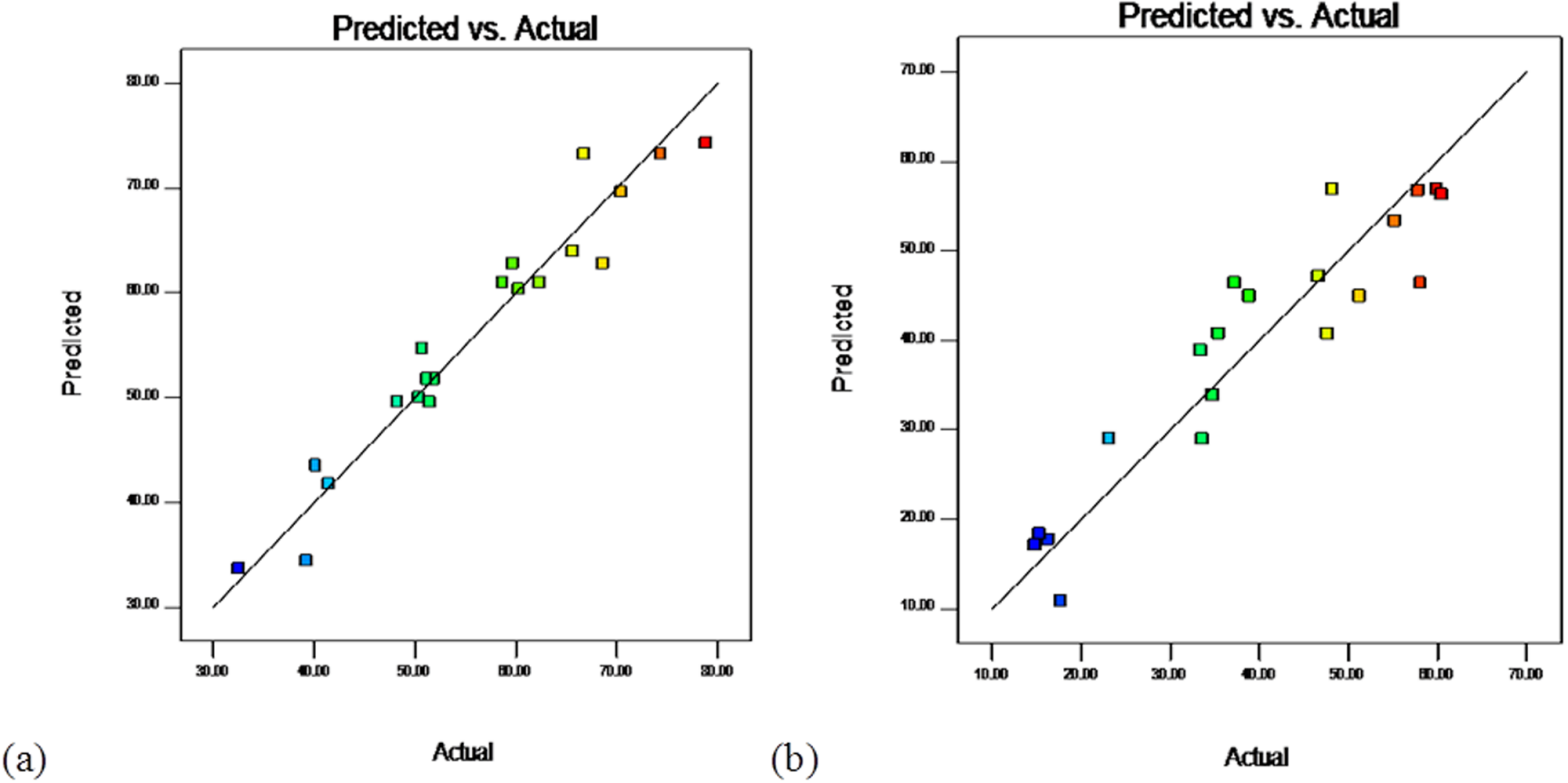
Plot of actual vs predicted values (a) COD (b) TOC removal efficiency.

The actual values versus predicted values obtained by Eqs (4) and (5) are shown in Fig 3(a) and 3(b). The plot of the correlation between the actual and predicted values for COD removal (*Y*_*1*_) suggested sufficient agreement between the real data and the data obtained from the model (Fig 3). Additionally, AP values higher than four (Table 3) for all of the responses substantiate that all of the predicted models can be employed to follow the design space defined by the CCD.

The other anticipated versus actual value plots for the TOC removal (*Y*_*2*_) were slightly different; this is shown in Fig 3b, which displays different removal efficiencies indicating the significance of the conformational and structural complexity of the landfill leachate toward photoelectrocatalytic oxidation.

### Removal efficiency of organic contaminants in landfill leachate

Under optimal conditions, a chromatogram presented no less than 73 kinds of organic elements, and the estimated concentrations in the landfill leachate from RO, which included 6 alkanes and 6 olefins, 6 aromatic hydrocarbons, 1 chlorinated hydrocarbon, 2 nitriles, 4 hydroxybenzene pollutants, 6 acids, 12 esters, 8 alcohols, 1 aldehyde, 9 ketones, 9 amides and large amounts of aromatic compounds, such as pyrene, phenanthrene, fluoranthene, fluorene, dimethyl phthalate, ethyl 2-thiolpropanoate, diisobutyl phthalate. Dimethyl phthalate and dibutyl phthalate are known as phthalic acid esters (PAEs), which are widely used in the plastic industry and are classified as endocrine disrupting chemicals (EDCs), interfering with the function of hormones in the human body, even at trace levels[32, 33], indicating the high toxicity level of the leachate. Many of these organic compounds are priority environmental pollutants as defined by the US Environmental Protection Agency and have carcinogenic and mutagenic properties[34].

As shown in Table 5, 38 organic materials were eliminated completely, the densities of 5 organic compounds were decreased by more than 80% and the elimination efficiencies of an additional 16 organic compounds were over 50%. The efficient removal of these compounds decreased the harmful effect on the receiving watercourse. These results coincide with the COD elimination efficiency. However, only 60.49% TOC removal was achieved by the photoelectrocatalytic process, indicating that decomposition and oxidation were not the primary removal route of hazardous organic contaminants in the PEC procedure. However, the density of organic contaminants in the landfill leachate was considerably reduced by the photoelectrocatalytic process.

**Table 5:** Concentration of organic micropollutants detected in the leachate in PEC treatment effluents.

No.	Organic Compounds	Raw Leachate	Optimization Photoelectrocatalysis	
			Residual	Removal efficiency (%)
1	trans-3-Hexenol	929.7	469.5	49.5
2	2,2'-Azobis(isobutyronitrile)	853.5	N.D.	100
3	5-Methyl-5-isopropyl-3-heptyne -2,6-dione	499.8	N.D.	100
4	Dodecane	958.6	144.7	84.9
5	methyl 6-oxoheptanoate	728.5	N.D.	100
6	Methyl(2,2-dimethylcyclohexyl)Ketone	856.3	726.1	15.2
7	syn-Tricyclo[4.2.1.12,5]dec-3-en-9-on	856.9	N.D.	100
8	8(9)-Hydroxy-Tricyclo[5.2.1.0(2,6)]Dec-3—Ene	966.8	N.D.	100
9	3,3a,4,6,7,7a-hexahydro-5H-4,7-methanoinden-5-one	946.1	N.D.	100
10	2-(1-Methyl-1H-imidazol-2-ylsulfanyl) -ethylamine	868.5	N.D.	100
11	N,N,2,6-tetramethyl-4-(1-oxidopyridin-4-yl)diazenyl-aniline	825.8	N.D.	100
12	2-Hydroxy-1,1,10-trimethyl-6,9-epidioxydecalin	855.2	N.D.	100
13	1,2,4-Oxadiazole,3-(4-methylphenyl)-5-(2,3,3-trifluoro-2-propenyl)	858.1	N.D.	100
14	Isobutyric acid 2-ethyl-3-hydroxyhexyl ester	931	N.D.	100
15	Dicyclopentadiene diepoxide	906	518.232	42.8
16	Dimethyl phthalate	993	800.358	19.4
17	3-ethoxy-3,7-dimethylocta-1,6-diene	847.1	N.D.	100
18	Methyl 3-(Boc-aMino)-3-(3-hydroxyphenyl)propanoate	876.2	N.D.	100
19	2-hydroxymyristic acid	871.1	N.D.	100
20	Ethyl 2-thiolpropanoate	809.6	N.D.	100
21	1-(2-aminoethyl)-3-phenylthiourea	824.2	N.D.	100
22	5-(4-Bromophenyl)-4,5-dihydro-1,3-oxazol-2-amine	862.1	N.D.	100
23	2-Dodecen-1-ylsuccinic Anhydride	901.2	N.D.	100
24	Benzyl N-acetyl-4,6-Benzylidenemuramic acid	864.4	N.D.	100
25	3-Deoxy-17beta-estradiol	856	227.7	73.4
26	4-Hydroxy-6-(4-methylphenyl)-2H-pyran-2-one	838.3	N.D.	100
27	3-[4-[(5-nitrothiazol-2-yl)azo](2-phenylethyl)amino]propiononitrile	881.2	N.D.	100
28	N-[6-(2-hydroxyethylsulfamoyl)naphthalen-2-yl]acetamide	781.2	N.D.	100
29	5β-Cholestan-3-one ethylene acetal	788.4	N.D.	100
30	2-Hexanone, 4-hydroxy-5,5-dimethyl-, (4S)- (9Cl)	815.3	465.5	42.9
31	Methyl octadeca-6,9-diynoate	857.2	N.D.	100
32	2,4,5,6,7,7a-Hexahydro-3,6-dimethyl-α-methylene-2-oxo-6-vinyl-5-benzofuranacetic acid methyl ester	908.3	N.D.	100
33	[[(3aS,9bβ)-3,3aβ,4,5,6,6a,7,8,9a,9bβ-Decahydro-6a-hydroxy-9aα-methyl-3-methylene-2,9-dioxoazuleno[4,5-b]furan -6α-yl]methyl]2-methylpropanoate	861.1	86.9	89.9
34	Ethyl-5-(4-bromophenyl)-isoxazole-4-carboxylate	875.4	N.D.	100
35	2-(2-Methylpropyl)pyrrolidine	866.4	N.D.	100
36	1-Heptatriacotanol	900.1	N.D.	100
37	6-Methyl-2-phenyl-quinoline	868.2	39.9	95.4
38	Phorbol	861.4	N.D.	100
39	cis-9,10-Epoxyoctadecanamide	862.3	N.D.	100
40	c2-oxo-3-tert-butyloxycarbonylamino-7-thia-1-azabicyclo(4.3.0)nonane-9-carboxylic acid	886.2	N.D.	100
41	(1aR)-1aα,1bβ,4,4aβ,5,7aα,7b,8,9,9a-Decahydro-3-hydroxymethyl-1,1,6,8α-tetramethyl-1H-cyclopropa[3,4]benz[1,2-e]azulene-5β,7bα,9β,9aα-tetrol 5,9,9a-triacetate	853.4	N.D.	100
42	Stigmastane-3,6-dione	871.4	N.D.	100
43	(Z)-9-Octadecenoic acid 3-(octadecyloxy)propyl ester	849.1	N.D.	100
44	1-(quinolin-5-yl)-1,2,3,4-tetrahydro-β-carboline	840.1	N.D.	100
45	2,3-di(docosanoyloxy)propyl docosanoate	824.3	N.D.	100
46	cis-11-Eicosenamide	846.2	402.8	52.4
47	12β-(Acetyloxy)-3β,8,14β-trihydroxypregn-5-en-20-one	727.2	403.6	44.5
48	β-carotene	712.1	N.D.	100
49	3,5-Di-tert-butylcatechol	778.4	N.D.	100
50	Triacetonamine	853.3	257.7	69.8
51	5-Octen-2-one, 3,6-dimethyl-	969.1	507.8	47.6
52	2,6,10-trimethyltetradecane	909.1	538.2	40.8
53	1-Chlorooctadecane	864.2	229.9	73.4
54	2-Allylphenol	836.1	252.5	69.8
55	Artemisic acid	841.3	281.8	66.5
56	Diisobutyl phthalate	985.1	142.8	85.5
57	1-Chloroeicosane	853.6	297.1	65.2
58	3-hydroxy-3-(4-Methoxyphenyl)-2,2-diMethyl-3-phenylpropanoic acid	865.4	591.1	31.7
59	N-(2-Naphthyl)aniline	956.7	305.2	68.1
60	Bisphenol A	807.1	538.3	33.3
61	Oleamide	974	619.5	36.4
62	3-ethyl-5-(2-ethylbutyl)octadecane	883.2	297.6	66.3
63	Quassin	768.1	149.0	80.6
64	Tetramethylphenol	909.8	584.1	35.8
65	Pyrene	404.4	264.1	34.7
66	Phenanthrene	728.3	570.3	21.7
67	Fluoranthene	343.3	260.9	24
68	Fluorene	45.8	35.0	23.5
69	Benzenemethanol	79.4	41.2	48
70	Cedrol	88.5	62.3	29.6
71	Thiophene	52.4	33.9	35.3
72	Octadecanoic	28.8	7.8	72.9
73	Acenaphthene	20.4	4.6	77.4

N.D. not detected.

Unit: μg/l

## Conclusions

The PEC process is an effective procedure for treating the dense leachate rejected from reverse osmosis. A mixture of three factors, five degrees CCD and response surface methodology were favorably used in the study to evaluate the individual and reaction effects of the parameters of the first pH, initial COD density and potential bias of the reaction mixture on the photoelectrocatalytic deterioration of landfill leachate using Cu/N co-doped TiO_2_(Ti). These methods were then used to ascertain the ideal conditions. The results indicate that the two factors of potential bias and initial COD concentration weighed in this study contributed a significant part in the elimination efficiency of COD and TOC. This study reveals that potential bias and initial COD concentration are more efficient than initial pH for the elimination of COD and TOC. For a photoelectrocatalytic remedy in which COD and TOC are a significant concern, both the potential bias and the COD concentration should be deliberated, with an adjustment between the elimination efficiencies of COD and TOC, and with the necessary pH.

By applying RSM, the ideal area for the reactor procedure was identified. The ideal environments obtained were an initial pH of 10.0, an initial COD concentration of 4377.98mgL^-1^ and 25.0 V of potential bias. Under the ideal conditions, the elimination efficiency of COD and TOC approached 67.0% and 82.5%, respectively, which were the best experimental points determined in the region of experimentation. The outcome of the confirmation test complied with the model predictions. The results demonstrate that RSM is useful to determine the most important operating components and ideal levels with the least amount of time and effort.

## Supporting information

S1 Data Table A COD removal efficiency at different pH and different COD concentration. (DOCX)
