# Supplementary material for: Optimization of the photoelectrocatalytic oxidation of landfill leachate using copper and nitrate co-doped TiO2 (Ti) by response surface methodology
Source: PLoS One. 2017 Jul 3;12(7):e0171234. doi: 10.1371/journal.pone.0171234 (PMC5495208; doi:10.1371/journal.pone.0171234)
Supplement: S1 Data — (DOCX) [file pone.0171234.s001.docx]

Dear editor,

According the request of PLoS ONE, we now upload four data sets underlying the findings in our study.

Table 1 COD removal efficiency at different pH and different COD concentration

| COD Removal efficiency (%) | pH=2  COD=2627mg/L  U=15V | pH=2  COD=2627 mg/L  U=5 | pH=4  COD=3503 mg/L  U=20 | pH=4  COD=1752 mg/L  U=10 |
| --- | --- | --- | --- | --- |
| 0min | 0 | 0 | 0 | 0 |
| 30min | 36.1 | 17.7 | 37.4 | 35.1 |
| 60min | 56.41 | 24.4 | 55.7 | 38.3 |
| 90min | 67.9 | 42.7 | 59.6 | 47.2 |
| 120min | 71.7 | 44.8 | 65.5 | 55.5 |
| 150min | 78.8 | 48.2 | 70.5 | 60.2 |
